# Supplementary material for: Learning curve and surgical outcome of robotic assisted colorectal surgery with ERAS program
Source: Sci Rep. 2022 Nov 29;12:20566. doi: 10.1038/s41598-022-24665-w (PMC9709162; doi:10.1038/s41598-022-24665-w)
Supplement: Supplementary file 1 — Supplementary Information 1. [file 41598_2022_24665_MOESM1_ESM.zip › LSC Raw data-Ñ[▒K/129/P_ñw╝╨░O▒Kñσ.pdf]

[redacted]

Pathologic diagnosis:
Colon, and terminal ileum, right hemicolectomy --- Tubulovillous adenoma with low grade dysplasia. The ileocecal valve, bilateral cut ends and mesocolic lymph nodes (0/19) are free of tumor.

Ancillary study for diagnosis:
Deep cut done on section D and E.

Gross description:
The specimen consists of a segment of colon, 17 cm in length, and 8.5 cm in proximal circumference, 10.5 cm in distal cut end circumference, and a segment of terminal ileum, 8.5 cm in length, and 4 cm in cut end circumference. No appendix is noted. A sessile polyp, 2x1 cm, is noted 8 cm to the nearest cut end. The ileocecal valve, and both cut ends are free of tumor. Representative part for section: A) proximal cut end B) distal cut end C) ileocecal valve D-E) polyp F-H) lymph nodes.

#T-59420_2 #M-82630_2 0 1105 000000
#T-C4560_2 #M-09183_2 0 1105 000000
#T-59200_2 #M-09183_2 0 1105 000000

已電子簽章

敬告：本單僅供醫師參考使用，不具任何申請效力！！
請病患妥為保管，若不慎遺失需自負個人資料外洩責任！
